# Synopsis of the myrmecophilous genus *Ecitonides* Wasmann (Coleoptera, Staphylinidae, Paederinae), with description of two new species from Brazil

**DOI:** 10.3897/zookeys.1267.175767

**Published:** 2026-01-30

**Authors:** Amanda Montanhini, Dagmara Żyła, Gabriel Biffi

**Affiliations:** 1 Universidade de São Paulo, Museu de Zoologia da Universidade de São Paulo, 04263-000, São Paulo, SP, Brazil Museu de Zoologia da Universidade de São Paulo São Paulo Brazil; 2 Museum of Nature Hamburg, Leibniz Institute for the Analysis of Biodiversity Change, Hamburg, Germany Leibniz Institute for the Analysis of Biodiversity Change Hamburg Germany

**Keywords:** Army ants, morphology, myrmecophily, neotropics, new species, taxonomy

## Abstract

The Neotropical genus of myrmecophilous beetles, *Ecitonides* Wasmann, 1894 (Staphylinidae: Paederinae: Lathrobiini), currently includes nine valid species distributed in French Guiana, Suriname, Brazil, Peru, Paraguay, and Argentina. Members of this genus are notable for the tuberculate ornamentation on the head, thorax, and abdomen. Although little is known about the biology of these beetles, there are records of association with different species of army ants (*Labidus* Jurine, 1807 and *Nomamyrmex* Borgmeier, 1936). In this study, a detailed comparative morphological study of all available species for study was conducted, descriptions of two new species, *Ecitonides colossus***sp. nov**. and *Ecitonides splendidus***sp. nov**., are provided, and lectotypes for *Ecitonides brevicornis* Wasmann, 1900 and *Ecitonides spectabilis* Borgmeier, 1932 are designated. Diagnoses, illustrations, and photographs are given; an identification key is provided for all 11 species as well as new geographic distribution data. The aim of this study is to provide morphological data that will serve as a tool for a future investigation of the phylogenetic position of the genus and facilitate species recognition through diagnostic morphological characters and an identification key.

## Introduction

The subfamily Paederinae Fleming, 1821 is among the most diverse groups within Staphylinidae, especially in the tropics, and includes approximately 8,000 described species worldwide (Żyła et al. 2022; Guzman et al. 2024; Newton 2025). Myrmecophily has evolved in some staphylinid subfamilies, such as Paederinae, and species of this subfamily associated with ants tend to be morphologically distinctive, often exhibiting unique and unusual features related to their symbiotic lifestyle, suggesting a high level of integration into ant colonies (Seevers 1965; Parker 2016).

*Ecitonides* Wasmann, 1894 (Staphylinidae: Paederinae: Lathrobiini) is a Neotropical genus of myrmecophilous rove beetles (Staphylinidae) associated with army ants of the genera *Labidus* Jurine, 1807 and *Nomamyrmex* Borgmeier, 1936. The genus comprises so far nine valid species: *E. tuberculosus* Wasmann, 1894, *E. brevicornis* Wasmann, 1900, *E. longiceps* Wasmann, 1900, *E. fraterculus* Borgmeier, 1959, *E. spectabilis* Borgmeier, 1932, *E. verrucosus* Bruch, 1933, *E. volans* Assing, 2012, *E. soesilae* Makhan, 2021 and *E. constanceae* Jenkins Shaw et al., 2023, distributed in Argentina, Brazil, Paraguay, Peru, and Suriname (Assing 2012; Jenkins Shaw et al. 2023).

The systematic position of *Ecitonides* is uncertain, and several hypotheses have been proposed regarding its relationships, including potential close genera and tribal affiliations. Wasmann (1894, 1900) placed *Ecitonides* near *Echiaster* Erichson, 1839, distinguishing it by the elongate-cylindrical head, forebody sculpture, and elongated fifth tarsomere. Blackwelder (1944) provisionally placed the genus, with other related genera, near *Myrmecosaurus* Wasmann, 1909, as did Bruch (1933). Seevers (1965) considered the relationship between *Ecitonides* and *Myrmecosaurus* as questionable, suggesting that the genera of the “*Ecitonides* group” (*Ecitonides*, *Bolbophites* Fauvel 1904, *Synecitonides* Reichensperger, 1936, *Labidophites* Borgmeier, 1956, *Ecitotropis* Borgmeier, 1936, *Ecitobium* Wasmann, 1923 and *Ecitosaurus* Fischer, 1943) may belong to Echiasterina, although he did not conduct any formal analysis to test this hypothesis. Assing (2012) considered that the genus might belong to the subtribe Cryptobiina (tribe Paederini) based on the morphology of the mouthparts, antennae, and male sexual characters. However, Schomann and Solodovnikov (2017) placed *Ecitonides* and related genera in the tribe Lathrobiini and suggested that members of the *Ecitonides*-group may be related to the ((Astenina + Stilicopsina) + (Medonina + Stilicina)) clade, based on published data and their examination of *Echiaster*. Recently, Jenkins Shaw et al. (2023) recovered *Ecitonides* within the “Medonina and allied taxa” clade of Lathrobiini, as the sister genus to *Ruptor* Żyła et al., 2022.

Species of *Ecitonides* share a robust body with prominences (tubercles) arranged in unequal rows over the body, an elongate head, an elongate scape, the presence of a constriction on protibiae, followed by a comb of golden setae and a tuft of long black or golden setae on the last abdominal segments. *Ecitonides* bears some resemblance to other myrmecophilous paederine genera such as *Bolbophites* and *Synecitonides* but can be easily distinguished from these by the more robust body, shorter appendages. and extreme development of the tubercles (Seevers 1965).

Although *Ecitonides* was revised by Borgmeier (1949) and later briefly revisited by Seevers (1965), most species, especially the earliest described, lack proper diagnoses, illustrations and/or photographs, and description of the genital structures. Moreover, very little is known about their biology and specimens are rarely collected, making it difficult to access information and provide clear guidance for morphological comparison and infer their sister-group relationships. In order to standardize the description of some characters, and introduce new morphological data for the genus, we provide new diagnoses, photographs, and illustrations for several species. In addition, two new species of *Ecitonides* are described and an updated identification key is given.

## Materials and methods

The studied specimens are deposited in the following collections: Museu de Zoologia da Universidade de São Paulo (**MZSP**, São Paulo, Brazil, S. Casari), Coleção Entomológica de Mato Grosso Eurides Furtado, Universidade Federal de Mato Grosso (**CEMT**, Cuiabá, Brazil, F. Vaz-de-Mello), Coleção Zoológica do Maranhão (**CZMA**, Caxias, Brazil, F. Limeira de Oliveira), Naturhistorisches Museum Wien (**NMW**, Vienna, Austria, H. Schillhammer) and Natural History Museum (**NHMUK**, London, Great Britain, M. Geiser and M. Barclay). Data of type specimen labels are cited verbatim (with original spelling retained) and are provided inside quotation marks (“ ”). The double slash (//) separates individual labels on each specimen.

Despite our several attempts, we were unable to access photographs of some of Wasmann’s type specimens supposedly deposited at the Natuurhistorisch Museum Maastricht (Maastricht, Netherlands). However, the MZSP houses part of the Borgmeier’s world-class entomological collection, which holds specimens of most of the species described by Wasmann, including some type specimens. These specimens were identified by Borgmeier himself by direct comparison with the holotypes, and diagnoses based on them were provided by Borgmeier (1949). Thomas Borgmeier was a German-Brazilian priest and entomologist, renowned for his contributions and extensive scientific output to the study of Neotropical ants, scuttle flies (Phoridae) and myrmecophilous Coleoptera and his role as founder and editor of specialized journals such as Revista de Entomologia and Studia Entomologica.

Specimens were photographed using a Canon EOS 6D Mark II camera equipped with a Canon MP-E 65 mm f/2.8 1–5× macro lens. For dissection, specimens were soaked in an aqueous solution of 10% KOH for 15 min to more than a day for relaxing, in some cases the solution was heated to 100°–150 °C, then immersed in acetic acid and water for neutralization. Dissected pieces were preserved on semi-permanent slides with jelly glycerin and coverslip for photographs and illustrations. A Zeiss Axioskop microscope with an attached Axiocam camera was used for photographs of dissected parts. Multifocal images were processed using Zerene Stacker software (v. 1.04) (Zerene systems LLC, Richland, Washington, United States of America). Scanning electron microscopy images were taken from mounted specimen using a Hitachi TM4000Plus Tabletop Microscope at the Museum of Nature Hamburg, Leibniz Institute for the Analysis of Biodiversity Change (ZMH, LIB). Illustrations were made under the same microscope and subsequently vectorized in Adobe Photoshop 21.0.2. The terminology used for morphological study and measurements follows mostly Assing (2012) and Jenkins Shaw et al. (2023). Measurements were taken in millimeters using ImageJ (Schneider et al. 2012) and the following measurements were taken, including the tubercles: Total body length (**TL**, from the anterior margin of the frons to the tip of the abdomen), head length (**HL**, from the anterior to posterior margins of the head), head width (**HW**, maximum width), pronotum length (**PL**, from the anterior to posterior margin), pronotum width (**PW**, maximum width), elytral length (**EL**, from the humeri to the posterior margin), elytral width (**EW**, maximum width) and length of the aedeagus (**LA**, from the base to the apex of the median lobe). The distribution map was generated using QGIS v. 3.32.0, based on locality data from specimen labels and additional records obtained from the literature.

## Results

### Taxonomy

**Family Staphylinidae Latreille, 1802**

**Subfamily Paederinae Fleming, 1821**

**Tribe Lathrobiini Laporte, 1835**

**Subtribe Incertae sedis**

#### Ecitonides

Taxon classificationAnimaliaColeopteraStaphylinidae

Wasmann, 1894

21893A8A-796E-50C1-BC2D-0A1D177B3DD7

Figs 1, 2, 3, 4, 5, 6, 7, 8, 9, 10, 11, 12, 13, 14, 15

Ecitonides Wasmann, 1894: 212 (original description). Wasmann 1900: 248; 1909: 182 (identification key); Blackwelder 1944: 128 (catalog); Borgmeier 1949: 121 (notes, redescription, and identification key); Seevers 1965: 317 (systematics); Asenjo et al. 2013: 280; 2019: 279 (catalog); Jenkins Shaw et al. 2023: 191 (phylogeny, identification key).

##### Type species.

*Ecitonides tuberculosus* Wasmann, 1894 (by monotypy).

##### Emended diagnosis.

In addition to the diagnosis provided by Seevers (1965), species of *Ecitonides* can be recognized by their robust body with dense, tuberculate sculpture, elongate head, stout appendages with a constriction on protibiae followed by a comb of golden setae, and the last abdominal segments with a tuft of long, black setae. *Ecitonides* can be distinguished from the morphologically similar genera *Bolbophites* and *Synecitonides* by wider, feebly converging posterior head margin with rounded angles, and by the head and pronotum with larger, asymmetrical, densely distributed setigerous tubercles (the other genera with smaller, regularly arranged or absent tubercles).

##### Description.

Male.

***Head***: elongate, ~ 1.5–3.0× longer than wide, oblong or rectangular; postocular region elongate, with lateral margins weakly converging posteriorly; dorsal surface entirely covered with tubercles, sometimes sparse, ventrally with or without tubercles. Eyes large or reduced, reniform, with short setae between ommatidia. Antennae (Fig. 9A–G) with 11 antennomeres, approximately same length or longer than head; antennomeres evenly pubescent; scape as long as antennomeres 2–4, 2–5 or 2–6 combined, with external or internal lateral margin sinuate, with one or two emarginations at apex; antennomeres 2–5 longer than wide, 6–9 either longer than wide or transverse, antennomere 11 longer than wide, apex tapering, often acute. Clypeus (clp) (Fig. 10A, B) transverse and membranous, sometimes bearing one pair of long, distal setae. Labrum (Fig. 10) well sclerotized, transverse, with anterior margin laterally emarginate (l.e), bearing long setae; anterior margin with 2–5 denticles (m.dt), with 4–6 longer setae, and one pair of smaller medial setae on either side of median denticle. Mandibles (Fig. 11E) asymmetrical, wider at base, narrowing apically, with one long lateral seta, one or two teeth and pore-like structures randomly distributed dorsally; prostheca (ps) reduced, restricted to basal portion, longer than wide, with thick setae at apex. Maxilla (Fig. 11A) with cardo elongate, dilated at apex; stipes (st) longer than wide, basistipes and ditistipes of subequal size; galea (gal) and lacinia (lac) stout and with distal lobe truncate, with densely setose comb, bearing multiple pore-like structures. Maxillary palpi (mx.p) with palpomere 1 short and longer than wide, glabrous; palpomere 2 longer than wide, wider apically, ~ 2× as long as 1, shorter than 3; palpomere 3 longest, fusiform, and densely setose; palpomere 4 shortest, aciculate, and glabrous. Labium (Fig. 11B), mentum and submentum separated by suture; prementum (p.m) longer than wide, with one M-shaped carina; ligula (lig) acute, with central sclerite; mentum transverse or trapezoidal, with one pair of long posterior setae, one pair of smaller lateral setae on each side and one basal row of smaller pores, larger pores in middle and multiple pores of variable sizes laterally near middle; submentum triangular, with anterior margins projected, fused with gula, with one pair of distal setae. Labial palpi (l.p) with palpomere 1 longer than wide, glabrous; palpomere 2 slightly widened medially, with few long apical setae; palpomere 3 small and aciculate, ~ 1/2 width of palpomere 2, glabrous. Gular sutures contiguous but not fused. Neck distinctive, 1/4 of head width.

**Figure 1.**
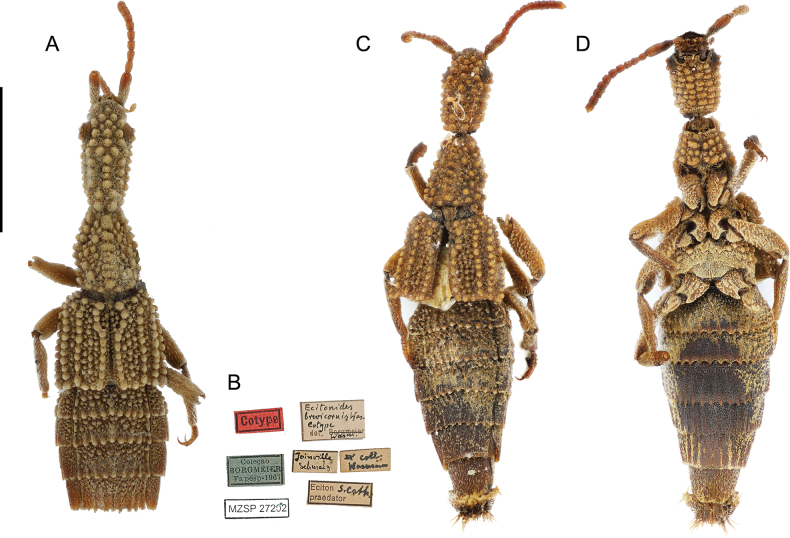
*Ecitonides brevicornis* Wasmann, 1900. **A, B**. Lectotype (MZSP 27202); **A**. Dorsal habitus; **B**. Labels; **C. D**. Dorsal and ventral habitus of a non-type specimen from Borgmeier’s collection (MZSP 21489). Scale bar: 2.0 mm.

**Figure 2.**
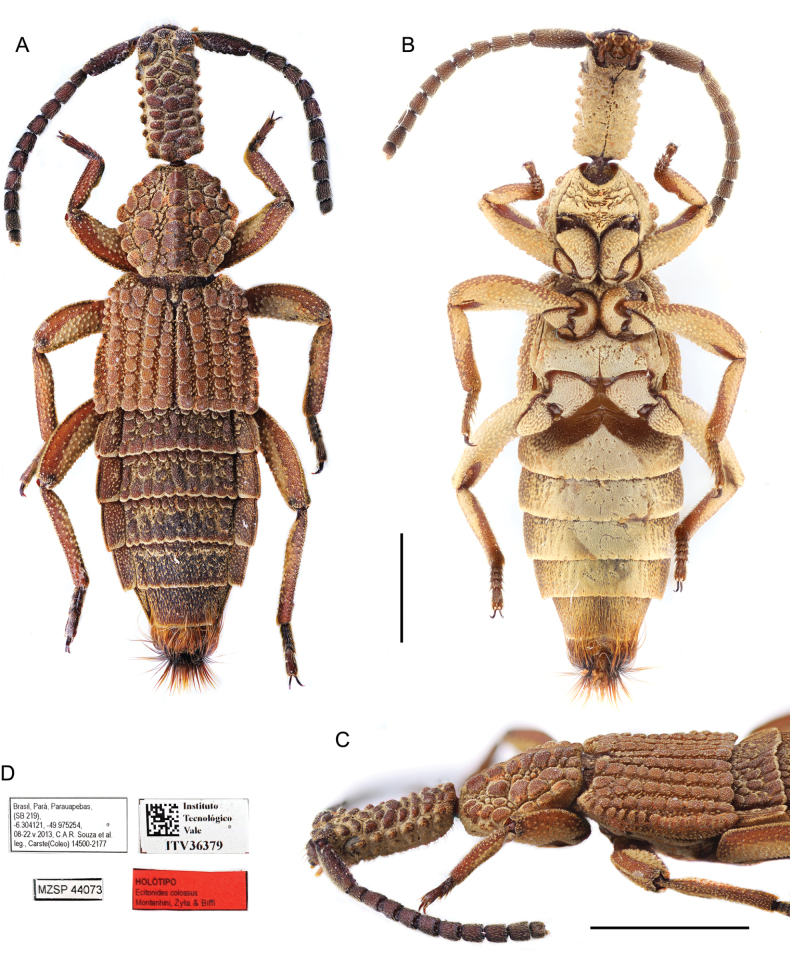
*Ecitonides colossus* sp. nov., holotype (MZSP 44073). **A**. Dorsal habitus; **B**. Ventral habitus; **C**. Head and thorax, lateral view; **D**. Labels. Scale bar: 2.0 mm.

**Figure 3.**
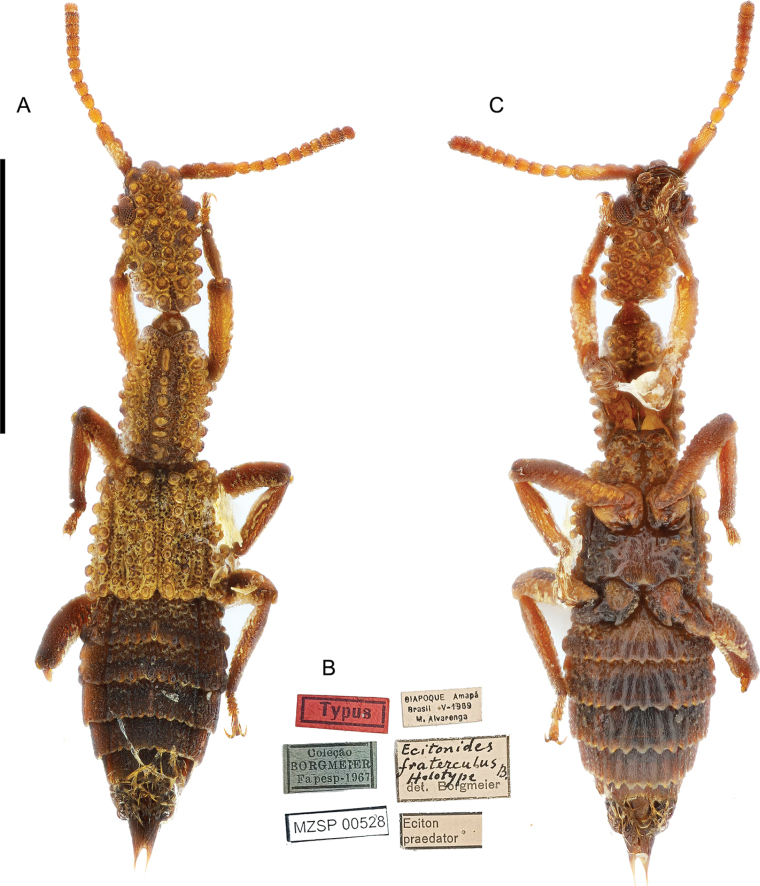
*Ecitonides fraterculus* Borgmeier, 1959, holotype (MZSP 00528). **A**. Dorsal habitus; **B**. Ventral habitus; **C**. Labels. Scale bar: 2.0 mm.

***Thorax***: pronotum shorter or nearly as long as head, longer than wide, subconical, trapezoidal or hexagonal, laterally and posteriorly sinuate due to tuberculate sculpture, anteriorly feebly sinuate; surface covered with rows of tubercles of variable sizes, forming slightly to strongly prominent median line. Prosternum with tubercles or with distinctive texture if tubercles absent; furcasternum triangular, with sharp longitudinal carina in middle, longer than tip of postprocoxal process, not reaching mesoventrite. Meso- and metaventrite with or without tubercles. Elytra wider or as wide as pronotum, each elytron with 3–5 rows of tubercles, laterally and posteriorly sinuate due to tuberculate sculpture; humeri slightly projected anteriorly. Membranous wings well developed. Legs stout, relatively long, covered with small tubercles and short, thick setae; prothoracic trochantins large, triangular and well exposed; coxae contiguous; procoxae subconical and projected, mesocoxae rounded, and metacoxae trapezoid and projected; femora enlarged, outer margins slightly convex, profemora with or without small dilatation near middle; protibiae with comb of golden setae on inner margin and one single longer seta (Fig. 14D), with small excavation near middle; mesotibiae with comb of golden setae, smaller and less dense than in protibiae; metatibiae with apical ctenidium on external side; tarsi 5-segmented (Fig. 9H–N), tarsomeres covered with long, golden setae dorsally, with or without long setae ventrally, with or without one dorsal median carina; tarsomere 1 nearly same size or longer than 2–4 combined, transverse or longer than wide; 2–4 subequal or slightly decreasing in size, transverse or longer than wide; tarsomere 5 longest, cylindrical, apically dilated, as long as or longer than 1–4 combined; claws long and unciform, with one basal tooth.

***Abdomen***: robust, narrowed towards apex, segments VII and VIII much narrower than others. Segments III–VI with pair of paratergites and VII with single paratergite on each side. Tergites III–VI and paratergites of III and IV with or without tubercles; VII with posterior margin straight, covered with small, thick setae; VIII posteriorly U-shaped and densely covered with short setae; IX and X with conspicuous tuft of long setae. Sternites III–VI with or without tubercles, with or without ridges extending beyond posterior margin; VII with posterior margin weakly emarginate medially and setose (Fig. 12A, B); VIII with deep U-shaped emargination (Fig. 12D, F); IX longer than wide with thick, apical setae (Fig. 12C). **Aedeagus** (Fig. 13) with median lobe varying from slender and slightly flattened to short and stout, bulbous at base, with narrow to truncate dorsal strut near foramen; apex of median lobe narrow and pointed or feebly rounded, internal sac with spine-like setae, laterally curved. Parameres absent.

**Female**. Similar to male, except for posterior margin of sternite VII straight and posterior margin of sternite VIII without deep emargination (Fig. 12B, E).

##### Measurements.

TL = 5.0–12.0.

##### Distribution.

*Ecitonides* is restricted to South America, recorded in Argentina, Brazil, French Guiana, Paraguay, Peru, and Suriname (Table 1, Fig. 15).

**Table 1.** Checklist of geographical records of *Ecitonides* species and their ant hosts species.

**Species**	**Hosts**	**Records (Country^1^: provinces/ states/ department^2^)**	**References**
*E. tuberculosus*	*L. praedator*	ARG: AR-N; BRA: MG, RJ, SC, SP; PRY: PY-3	Wasmann 1894, 1909; Borgmeier 1949; Asenjo et al. 2013; new data
*E. longiceps*	*L. coecus*	BRA: SC	Wasmann 1900; Asenjo et al. 2013
*E. brevicornis*	*L. praedator*	BRA: GO, ES, RJ, SP, SC	Wasmann 1900; Borgmeier 1949; Asenjo et al. 2013; new data
*E. verrucosus*	*L. coecus*	ARG: AR-N; BRA: GO	Bruch 1933; Borgmeier 1949; Asenjo et al. 2013; new data
*E. spectabilis*	*N. esenbeckii*; *N. hartiigi*	BRA: GO, MG, SP	Borgmeier 1932, 1949; Asenjo et al. 2013; new data
*E. fraterculus*	*L. praedator*	BRA: AP	Borgmeier 1959; new data
*E. volans*	Unknown	GUF; PER: PE-HUC	Assing 2012
*E. soesilae*	Unknown	SUR	Makhan 2021
*E. constanceae*	Unknown	PER: PE-MDD	Jenkins Shaw et al. 2023
*E. colossus* sp. nov.	Unknown	BRA: PA	new data
*E. splendidus* sp. nov.	Unknown	BRA: MA	new data

^1^Trigraph country codes: ARG: Argentina; BRA: Brazil; GUF: French Guiana; PER: Peru; PRY: Paraguay; SUR: Suriname. ^2^Acronyms of states, provinces, or departments: BRA: AP: Amapá, ES: Espírito Santo, GO: Goiás, MA: Maranhão, MG: Minas Gerais, PA: Pará, RJ: Rio de Janeiro, SC: Santa Catarina, SP: São Paulo; ARG: AR-N: Misiones; PER: PE-HUC: Huánuco, PE-MDD: Madre de Dios; PRY: PY-3: Cordillera.

##### Remarks.

The genus description is a synthesis of the morphological study of the *Ecitonides* specimens deposited at MZSP (majority), CEMT, CZMA, NMW, NHMUK, and information retrieved from the literature. Some terms used in previous descriptions are not directly comparable, and others are re-interpreted based on our morphological studies. In the case of *E. longiceps*, Wasmann (1900, 1909) described the head as 4× longer than wide, while later Borgmeier (1949) corrected the proportion to ~ 3× longer than wide. As the type specimens of *E. longiceps* were not available for our study and the illustration provided by Wasmann does not match the description, our redescription follows the proportions found by Borgmeier, and the approximate size observed in the illustration of the habitus (Wasmann 1900: taf. II, fig. 15).

### Identification key to species of the genus *Ecitonides*

This following key is partially based on the original descriptions, since holotypes of *E. longiceps*, *E. soesilae*, *E. verrucosus*, and *E. constanceae* were not assessed, and on previous keys provided by Wasmann (1900: 248), Borgmeier (1949: 122), and Jenkins Shaw et al. (2023: 191).

**Table:** 

1	Eyes small, barely visible in dorsal view (Fig. 2A)	**2**
–	Eyes large, easily visible in dorsal view (Fig. 1A)	**3**
2	Pronotum subconical, tarsomeres longer than wide (Wasmann, 1900: taf. II, fig. 15)	***E. longiceps***
–	Pronotum hexagonal, tarsomeres transverse (Fig. 2, 9H)	***Ecitonides colossus* sp. nov**.
3	Antennae approximately as long as head	**4**
–	Antennae approximately twice as long as head	**5**
4	Antennomeres 6–10 subquadrate (Fig. 9E)	***E. brevicornis***
–	Antennomeres 6–10 transverse (Fig. 9F)	***E. fraterculus***
5	Pronotum subconical	**6**
–	Pronotum slightly longer than wide to hexagonal or trapezoidal	**8**
6	Pronotum with 4 regular rows of tubercles, scape as long as antennomeres 2–6 combined (Fig. 6, 9D)	***E. tuberculosus***
–	Pronotum with 5 regular rows of tubercles, scape as long as antennomeres 2–5 combined	**7**
7	Tergites III–V with dense tuberculate sculpture, tubercles bulbous and very prominent (Fig. 8A)	***E. volans***
–	Tergites III–V with less dense tuberculate sculpture, tubercles slightly flattened	***E. soesilae***
8	Pronotum trapezoidal, head oval and wider posteriorly (Fig. 7A)	***E. verrucosus***
–	Pronotum slightly longer than wide or hexagonal, head more rectangular	**9**
9	Labrum with 3 denticles (Fig. 10B)	**10**
–	Labrum with 5 denticles (Fig. 10D)	***Ecitonides splendidus* sp. nov**.
10	Eyes larger, distinctly interrupting the outline of the head in dorsal view, tubercles on pronotum seemingly more flattened in dorsal view	***E. constanceae***
–	Eyes slightly smaller, not distinctly interrupting the outline of the head in dorsal view, tubercles on pronotum less flattened in dorsal view (Fig. 4A, B)	***E. spectabilis***

### Species descriptions

#### Ecitonides brevicornis

Taxon classificationAnimaliaColeopteraStaphylinidae

Wasmann, 1900

3304D2BC-432C-50A8-981B-531BB61C4774

Figs 1, 9 E, L, 10E, 13J, K, 14

Ecitonides brevicornis Wasmann 1900: 249. Borgmeier 1949: 122 (redescription and notes).

##### Type locality.

Brazil • Santa Catarina: Joinville, 26°18'14"S, 48°50'45"W, Schmalzmaier leg.

##### Type specimen examined.

***Lectotype*** (MZSP, present designation) male, dry pinned, with genitalia in a separate slide. Original labels: “Lectótipo, Ecitonides brevicornis Wasmann, 1900, designated by Montanhini, Żyła and Biffi, 2025” // “Brasil, Santa Catarina, Joinville, Schmalz.” // Eciton praedator” // “Ecitonides brevicornis cotype. det. Wasmann” // “Ex. Coll. Wasmann”// “Coleção Borgmeier FAPESP, 1967” // “MZSP 27202”.

**Figure 4.**
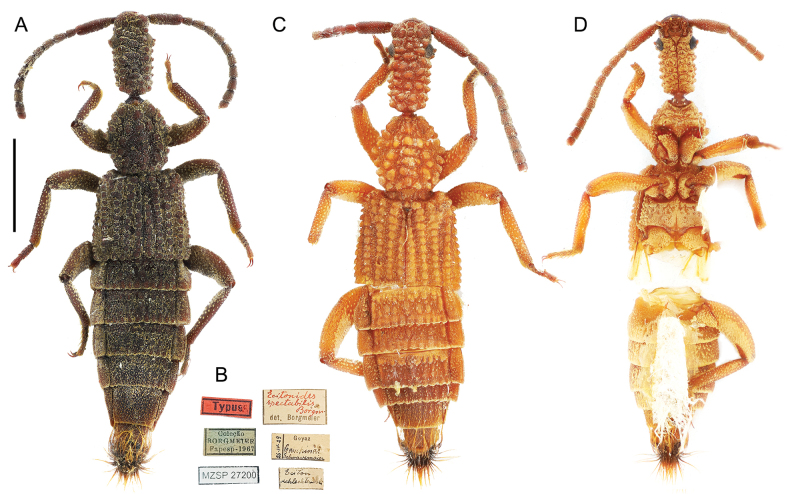
*Ecitonides spectabilis* Borgmeier, 1932. **A, B**. Lectotype (MZSP 27200); **A**. Dorsal habitus; **B**. Labels; **C, D**. Non-type specimen (MZSP 21499) showing the color variation within the species. Scale bar: 2.0 mm.

**Figure 5.**
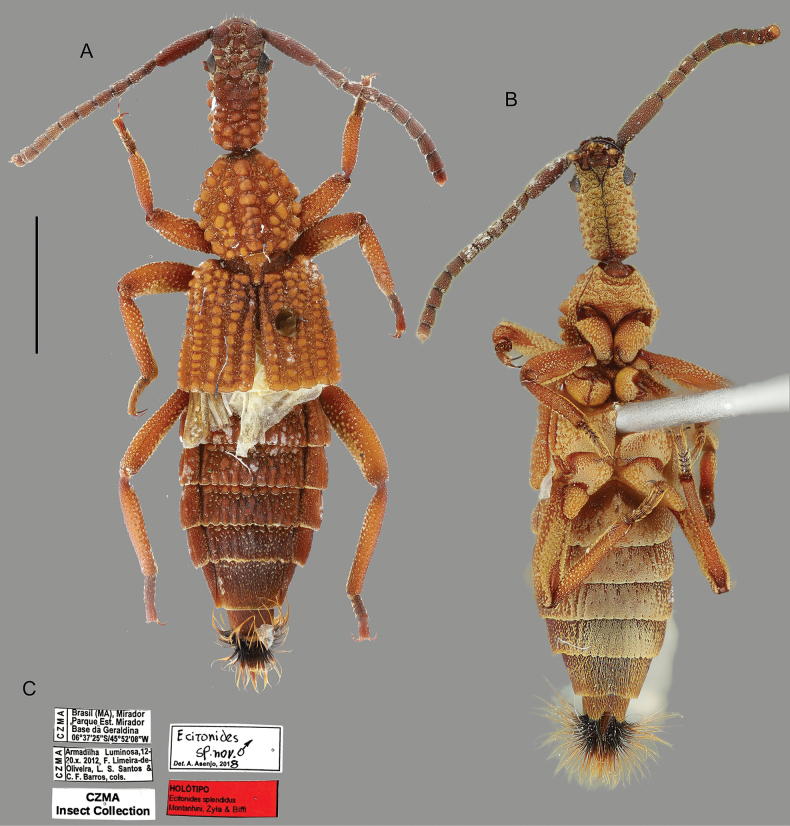
*Ecitonides splendidus* sp. nov., holotype (CZMA: FLO 15708): **A**. Dorsal habitus; **B**. Ventral habitus; **C**. Labels. Scale bar: 2.0 mm.

**Figure 6.**
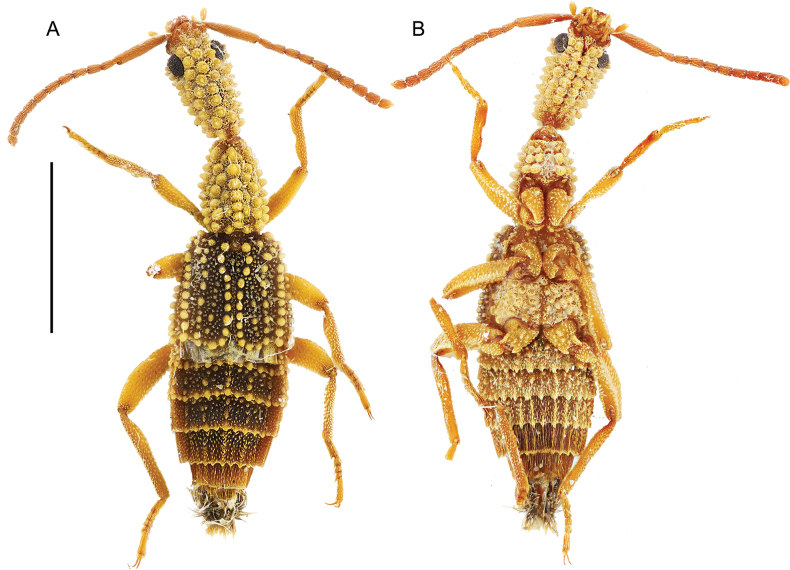
*Ecitonides tuberculosus* Wasmann, 1894, non-type specimen (MZSP 21511). **A**. Dorsal habitus; **B**. Ventral habitus. Scale bar: 2.0 mm.

##### Other material examined.

Brazil (MZSP) • 2 ♀♀; Goiás, Campinas; 19 Jul. 1937; Schwarzmaier leg.; MZSP 21488 • 2 undetermined sex; same collection data as for preceding; Jan. 1946; MZSP 21490 • 1 undetermined sex; same collection data as for preceding; 1 Dec. 1936; MZSP 21492 • 1 undetermined sex; Rio de Janeiro, Itatiaia; 1 Dec. 1935; J.F. Zikan leg.; MZSP 21491 • 1 ♀; São Paulo, Itú, Fazenda Pau d’Alho; 22 Jan. 1961; U. Martins leg.; MZSP 21493 • 1 ♀; São Paulo, Pindamonhangaba; Schwarzmaier leg.; MZSP 21489. BRAZIL (NMW) • 1 undetermined sex; Minas Gerais; 16 Mar. 1923. • São Paulo, Pindamonhangaba.

##### Diagnosis.

Body not very robust, head oval, pronotum longer than wide and subconical, and prominent tubercles over the body as in *E. fraterculus*, *E. longiceps*, *E. soesilae*, *E. tuberculosus*, and *E. volans*. Different from *E. fraterculus* by antennomeres 6–10 as long as wide (transverse in *E. fraterculus*) and by each elytron with four rows of tubercles (3 in *E. fraterculus*), from *E. longiceps* by larger eyes, from *E. tuberculosus* by antennae of same length as head (almost 2× longer than head in *E. tuberculosus*) and from *E. soesilae* and *E. volans* by pronotum with four rows of tubercles (5 rows in other two species).

##### Additional characters.

Antennae (Fig. 9E) with scape as long as antennomeres 2–5; antennomeres 2 and 3 subequal in length; antennomeres 2–5 longer than wide, 6–10 subquadrate, antennomere 11 slightly smaller than 10; labrum (Fig. 10E) with three denticles of subequal size; abdomen with tergites III–V and paratergites of segment III bearing small tubercles, VI bearing only impressions at base; sternites III–VI with ridges extending beyond posterior margin and few lateral tubercles. Aedeagus (Fig. 13J, K) with median lobe short and stout, with truncate dorsal strut near foramen, slightly narrowing towards apex, apex of median lobe rounded.

##### Measurements.

TL: 6.5–7.0; HL: 1.53; HW: 0.8; PL: 1.28; PW: 0.92; EL: 1.39; EW: 1.47; LA: 0.9.

##### Distribution.

BRAZIL, Goiás, Espírito Santo, Rio de Janeiro, São Paulo, and Santa Catarina (Fig. 15).

##### Host ant.

*Labidus praedator*.

##### Remarks.

Some of the studied specimens are old, entirely glued to cardboards, and therefore fragile. In order to preserve these specimens, it was decided not to unglue them, which made sex determination impossible. Wasmann (1900) did not designate a holotype or define the type locality of the species; he only described two specimens that were found in two localities, Teresópolis (Brazil, Rio de Janeiro) and Joinville (Brazil, Santa Catarina), and we did not have access to the other specimen from the type series deposited in Wasmann’s collection to confirm the conspecificity of the syntypes. The syntype deposited in Borgmeier’s collection (MZSP) originally belonged to Wasmann’s collection, as indicated by the label “Ex. Coll. Wasmann”, and is the same specimen Borgmeier (1949) mentioned as the “paratype” in his review, originating from Joinville (Brazil, Santa Catarina). The exact locality of the other specimen in the type series is unknown. Since Wasmann did not designate a holotype or a type locality, and given our inability to access the other syntype, we here designate the aforementioned specimen as a lectotype, thereby fixing the type locality for Joinville, Santa Catarina Brazil.

#### Ecitonides colossus sp. nov.

Taxon classificationAnimaliaColeopteraStaphylinidae

477586F4-BA2E-5CBF-A655-CC0D753AA0CC

https://zoobank.org/955F5C39-CD7A-48ED-A60B-EF1E2F229AEB

Figs 2, 9 A, H, 10C, 12C, F, 13A–C

##### Type locality.

Brazil • Pará: Parauapebas (SB 219), 6°18'14.84"S, 49°58'30.9"W, 08–22 May. 2013, Souza et al. leg.

##### Type specimen.

***Holotype*** (MZSP) male, dry pinned, with genitalia in a separate slide. Original labels: “Lectótipo, *Ecitonides colossus* sp. nov. Montanhini, Żyła and Biffi, 2025”// “Brasil, Pará, Parauapebas, (SB 219), -6.304121, -49.975254, 08–22.v.2013, C.A.R. Souza et al. leg., Carste (Coleo) 14500-2177” // “MZSP44073” // “Instituto Tecnológico da Vale, ITV 36379” // “MZSP 44073”.

##### Diagnosis.

Body robust, head rectangular, pronotum hexagonal, and flattened tubercles over the body as in *E. constanceae*, *E. spectabilis* and *E. splendidus* sp. nov. It differs from all other species by the eyes reduced in size, almost indistinct in dorsal view.

##### Description.

Body length 11.9 mm. Body dark reddish-brown dorsally and bright yellowish-brown ventrally; antennae, tarsi, and outer margins of tibiae and femora slightly darker than body. Head, pronotum, elytra, tergites III–V, and anterior margin of tergite VI covered with tubercles, ventral side with distinctive texture and without distinct tubercles.

***Head***: elongate, ~ 2× as long as wide (HL: 2.5; HW: 1.1), rectangular, dorsal surface flat and covered with tubercles arranged in irregular longitudinal rows; ventral surface with distinct texture, without tubercles and covered with short, sparse, yellowish setae; frons as wide as posterior region; posterior margin only slightly converging. Eyes reduced, grayish, weakly visible from above, occupying ~ 1/7 of lateral side of head (Fig. 2C). Antennae (Fig. 9A) elongate and rather robust, twice longer than head, reaching posterior margin of pronotum; antennomeres moderately pubescent; scape as long as antennomeres 2–5 combined, with external margin sinuate, with two apical emarginations; antennomeres 2–10 longer than wide; 2 shorter than 3; 3 longer than 4–10; 4–10 subequal in length; antennomere 11 shorter than 10, subquadrate, apex acute. Labrum (Fig. 10C) well sclerotized, transverse, slightly emarginate and with three denticles, median one pointed and one slightly rounded tooth on each side.

**Figure 7.**
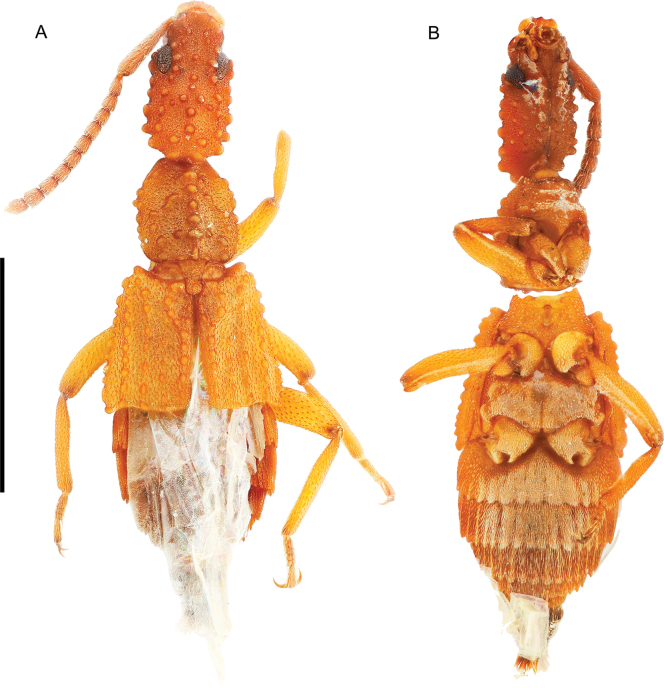
*Ecitonides verrucosus* Bruch, 1933, non-type specimen (MZSP 21500). **A**. Dorsal habitus; **B**. Ventral habitus; **C**. Labels. Scale bar: 2.0 mm.

**Figure 8.**
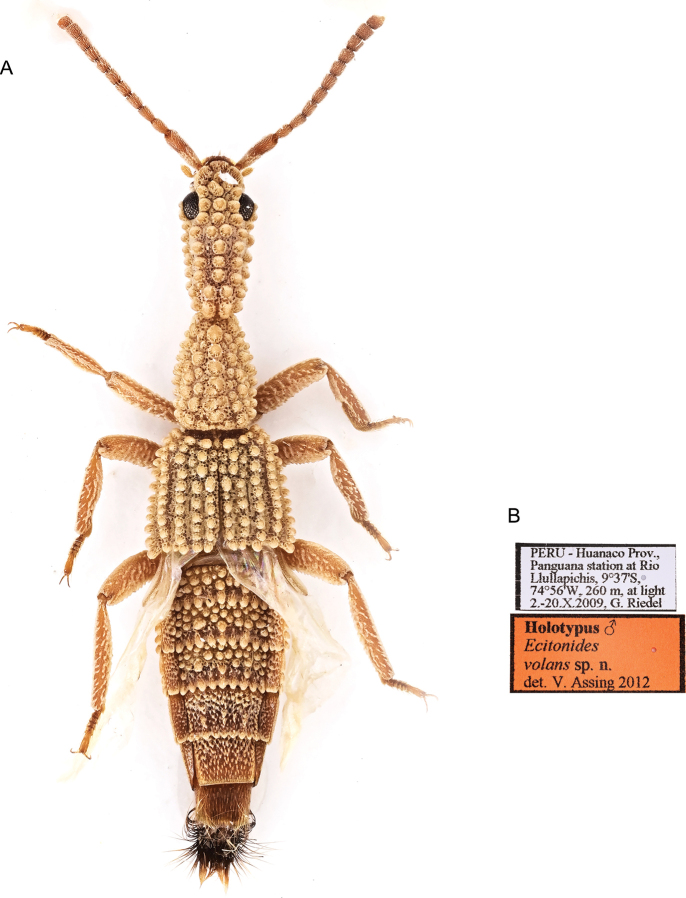
*Ecitonides volans* Assing, 2012, holotype (NMW). **A**. Dorsal habitus; **B**. Labels. Photos by Harald Schillhammer (NMW).

**Figure 9.**
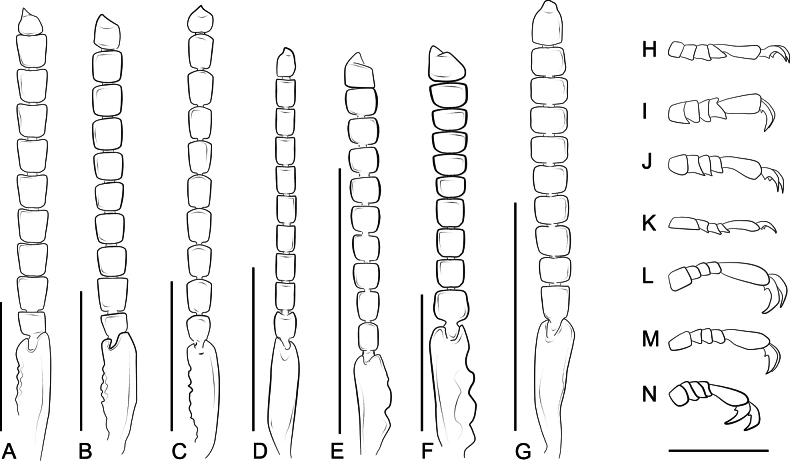
Antennae and tarsi of *Ecitonides* species. **A–G**. Antennae; **H–N**. Foretarsi. **A**, **H**. *Ecitonides colossus* sp. nov.; **B**, **I**. *E. splendidus* sp. nov.; **C**, **J**. *E. spectabilis*; **D**, **K**. *E. tuberculosus*; **E**, **L**. *E. brevicornis*; **F**, **M**. *E. fraterculus*; **G**, **N**. *E. verrucosus*. Scale bars: 1.0 mm (**A–G**); 0.5 mm (**G–M**).

***Thorax***: pronotum convex, hexagonal (PL: 4.6; PW: 2.2), widest at middle, wider than head; lateral margins diverging posteriorly at first half and converging posteriad and sinuate due to tuberculate sculpture; posterior margin sinuate and slightly wider than anterior margin. Pronotum covered with unequal rows of large tubercles interspaced with smaller tubercles; median tubercles forming slight longitudinal crest. Prosternum with distinctive texture, without distinct tubercles, hypomera bearing small tubercles. Elytra (EL: 2.5; EW: 3.0) wider than pronotum, sinuate laterally and posteriorly due to tuberculate sculpture, slightly wider at posterior 1/2; anterior margin declivous; humeri weakly projected; each elytron with five regular longitudinal rows of small tubercles; each row marginated by small thick setae. Meso- and metaventrite without conspicuous tubercles and bearing short, thick setae; metaventrite longer than mesoventrite; mesoventrite bearing small and shallow impression; mesosternal process acute and not reaching metasternal process; metasternal process small and truncate. Legs stout and relatively long, covered with small sparse tubercles and short thick setae; tarsomeres 1–4 transverse and subequal in size, 5 longest, cylindrical, apically dilated, slightly longer than previous four combined (Fig. 9H).

***Abdomen***: tergites III–V with posterior margin sinuate, more conspicuous in III and IV; VI with posterior margin slightly crenulated. Tergites III–IV with whole surface covered with tubercles and thick setae in between; tergite V with distinct row of tubercles only at base; VI without tuberculate sculpture, only impressions at base; VII with posterior margin straight, covered with small, thick setae; VIII medially U-shaped and densely covered with short, golden setae. Sternites III–VI with distinctive texture and covered with yellowish, short setae, laterally bearing thicker setae, outer margins weakly crenulated; VII covered with setae and slightly emarginate medially; VIII with median deep rounded emargination (Fig. 12F); IX longer than wide with apical long setae (Fig. 12C). **Aedeagus** (Fig. 13A–C) (LA: 1.6) long and slender, slightly flattened and feebly sinuate near apex, bulbous at base, with truncate dorsal strut near foramen; apex of median lobe narrow and pointed, laterally curved.

##### Etymology.

The specific epithet, a Latinized noun in nominative case derived from the Ancient Greek κολοσσός (kolossós), which means “large/giant statue”, in allusion to the larger and more robust body of the new species.

##### Distribution.

BRAZIL, Pará (Fig. 15).

##### Host ant.

Unknown.

#### Ecitonides fraterculus

Taxon classificationAnimaliaColeopteraStaphylinidae

Borgmeier, 1959

7104BEF6-0FA2-5779-9082-42FB521E3488

**Figure 10.**
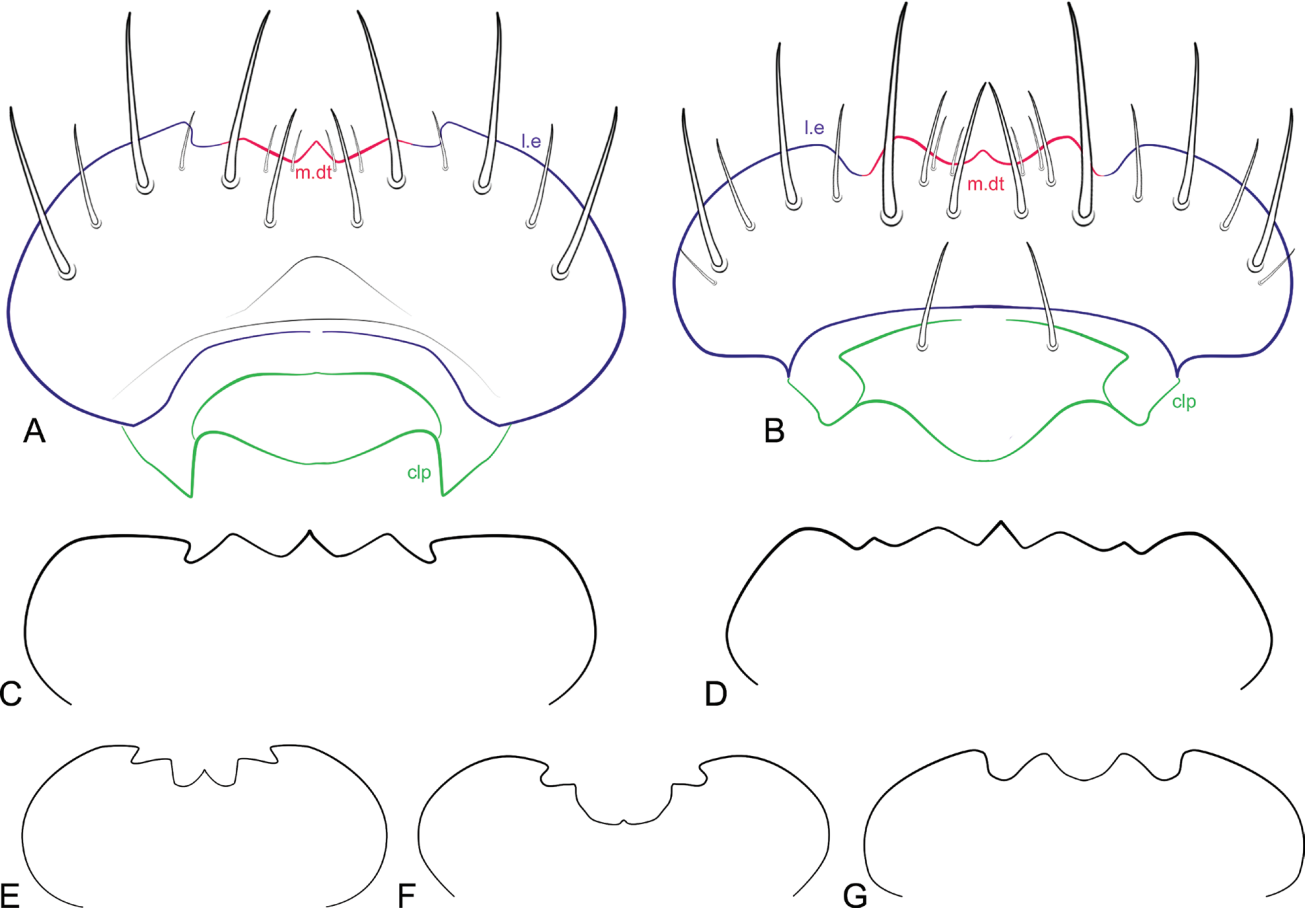
Labrum of *Ecitonides* species. **A**. *Ecitonides tuberculosus*; **B**. *E. spectabilis*; C. *E. colossus* sp. nov.; **D**. *E. splendidus* sp. nov.; **E**. *E. brevicornis*; **F**. *E. fraterculus*; **G**. *E. verrucosus*. Abbreviations: m.dt: medial denticles; l.e: lateral emargination; clp: clypeus. Figures not to scale.

Figs 3, 9 F, M, 10F, 13L, M

Ecitonides fraterculus Borgmeier 1959: 3.

##### Type locality.

Brazil, Amapá: Oiapoque, 3°49'29"N, 51°49'05"W, May. 1959, M. Alvarenga leg.

##### Type specimen examined.

***Holotype*** (MZSP) male, dry pinned, with genitalia in a separate slide. Original labels: “Typus” // “OIAPOQUE - Amapá, Brasil, V. 1959. M Alvarenga” // “*Eciton praedator*” // “*Ecitonides fraterculus* B. Holotype. det. Borgmeier.” // “Coleção BORGMEIER Fapesp - 1967” // “MZSP 00528” // “MZSP 00528”.

##### Diagnosis.

Body less robust, head oval, pronotum longer than wide and subconical, and prominent tubercles over the body as in *E. brevicornis*, *E. longiceps*, *E. soesilae*, *E. tuberculosus*, and *E. volans*. From *E. brevicornis*, it differs by antennomeres 6–10 transverse (as long as wide in *E. brevicornis*) and by each elytron with three rows of tubercles (4 in *E. brevicornis*), from *E. longiceps* by larger eyes, from *E. tuberculosus* by antennae of the same length as head (~ 2× longer than head in *E. tuberculosus*), and from *E. soesilae* and *E. volans* by each elytron with three rows of tubercles (5 rows in other two species).

##### Additional characters.

Antennae (Fig. 9F) with scape as long as antennomeres 2–6 combined; antennomeres 2 and 3 subequal in size; antennomeres 2–5 longer than wide; antennomeres 6–10 transverse, slightly increasing in width towards apex; antennomere 11 shorter than 10; labrum (Fig. 10F) with three denticles, median one deeply emarginate and lateral pair closer to lateral emargination than to median denticle. Abdomen with tergites III–V and paratergites of III and IV bearing small tubercles; sternites III–VI with ridges extending beyond posterior margin and few lateral tubercles. Aedeagus (Fig. 13L, M) with median lobe short and stout, with truncate and short dorsal strut; apex of median lobe somewhat wide rounded.

**Figure 11.**
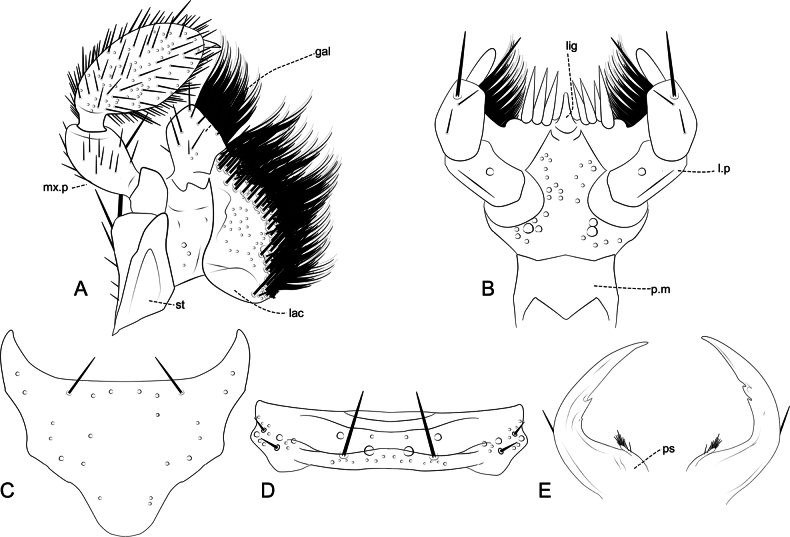
Mouthparts of *Ecitonides tuberculosus*. **A**. Maxilla; **B**. Labium, prementum; **C**. Labium, submentum; **D**. Labium, mentum; **E**. Mandibles. Abbreviations: mx.p: maxillary palpus; st.: stipes; lac: lacinia; gal: galea; p.m: prementum; l.p: labial palpus; lig: ligula; ps: prostheca. Figures not to scale.

**Figure 12.**
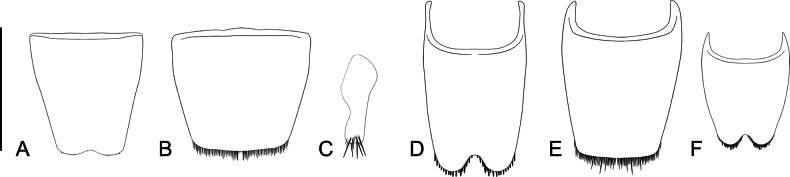
Abdominal sternites of *Ecitonides* species. **A**. *E. verrucosus*, sternite VII; **B**. *E. spectabilis*, sternite VII; **C**. *E. colossus* sp. nov., sternite IX; **D**. *E. verrucosus*, sternite VIII; **E**. *E. spectabilis*, sternite VIII; **F**. *E. colossus*, sternite VIII. Scale bars: 0.1 mm.

##### Measurements.

TL: 5.0; HL: 1.31; HW: 0.68; PL: 1.1; PW: 0.69; EL: 1.05; EW: 1.05; LA: 0.7.

##### Distribution.

BRAZIL, Amapá (Fig. 15).

##### Host ant.

*Labidus praedator*.

#### Ecitonides spectabilis

Taxon classificationAnimaliaColeopteraStaphylinidae

Borgmeier, 1932

591AA656-2841-58E8-8B17-213BE6449F27

Figs 4, 9 C, J, 10B, 12B, E

Ecitonides spectabilis Borgmeier 1932: 398. Borgmeier 1949: 124 (notes).

##### Type locality.

Brazil • Goiás: Goiânia (Setor Campinas), 16°39'38"S, 49°17'3"W, 26 Apr. 1929, Schwarzmaier leg.

##### Type specimens examined.

***Lectotype*** (MZSP, present designation) female, dry pinned. Original labels: “Lectótipo, *Ecitonides spectabilis* Borgmeier, 1932, designated by Montanhini, Żyła and Biffi, 2025” // “Typus” // Goyas, Campinas, Schwarzmaier, 26-IV-29” // “Ecitonides spectabilis Borg. det. Borgmeier.” // “*Eciton schlechtendali*” // “Coleção BORGMEIER, Fapesp - 1967” // “MZSP 27200” • ***Paralectotypes*** (MZSP, present designation) 3 females, dry pinned. Original labels: “Paralectótipo, *Ecitonides spectabilis* Borgmeier, 1932, designated by Montanhini, Żyła and Biffi, 2025” // “Typus” // “Goyas, Campinas, Schwarzmaier, XII-1929” // “*Ecitonides spectabilis* Borg. det. Borgmeier.” // “*Eciton crassicorne*” // “Coleção BORGMEIER, Fapesp - 1967” // “MZSP 27201” • “Paralectótipo, *Ecitonides spectabilis* Borgmeier, 1932, designated by Montanhini, Żyła and Biffi, 2025” // “Goyas, Campinas, Schwarzmaier, 15.V.1924” // “*Ecitonides spectabilis* Borg. paratype det. Borgmeier” // “*E. schlechtendali*” //“Coleção BORGMEIER, Fapesp - 1967” // “MZSP 21494” • “Paralectotype, *Ecitonides spectabilis* Borgmeier, 1932, designated by Montanhini, Żyła and Biffi, 2025” // “Goyas, Campinas, Schwarzmaier, 14.V.1924, *E. schlechtendali*” // “*Ecitonides spectabilis* Borg. paratype det. Borgmeier” //“Coleção BORGMEIER, Fapesp - 1967” // “MZSP 21495”.

##### Other material examined.

Brazil (all MZSP) • 1 ♀; Goiás, Campinas; Jan. 1934; Schwarzmaier leg.; MZSP 21497 • 1 ♀; Minas Gerais, Unaí, Fazenda Bolívia; 22–24 Dec. 1964; MZSP 21498 • 1 ♀; São Paulo, Barretos; May. 1935; O.S. Barcellos leg.; MZSP 21496 • 1 ♀; São Paulo, Itú, Fazenda Pau D’Alho; 13 Jan. 1962 • U. Martins leg.; MZSP 21499.

##### Diagnosis.

Body more robust, head rectangular, pronotum slightly longer than wide to hexagonal, and flattened tubercles over body as in *E. constanceae*, *E. colossus*, and *E. splendidus* sp. nov. From *E. constanceae*, it differs by smaller eyes (bigger and distinctly interrupting the outline of the head in dorsal view in *E. constanceae*), from *E. colossus* sp. nov. by bigger eyes, and from *E. splendidus* sp. nov. by hexagonal pronotum (slightly longer than wide in *E. spectabilis*).

##### Additional characters.

Antennae (Fig. 9C) ~ 1.5× longer than head with scape as long as antennomeres 2–5 combined; antennomere 2 shorter than 3, antennomeres 2–10 longer than wide and of subequal size; antennomere 11 shorter than 10; labrum (Fig. 10B) with three denticles, median denticle smaller and pointed, with two rounded larger denticles on each side; pronotum longer than wide, somewhat hexagonal; elytra with five rows of tubercles. Abdomen with tergites III–V bearing large flattened tubercles, VI bearing only impressions at base; sternites not bearing tubercles.

##### Measurements.

TL: 9.0–11.0; HL: 1.81; HW: 0.95; PL: 1.57; PW: 1.49; EL: 1.97; EW: 1.88.

##### Distribution.

BRAZIL, Goiás, Minas Gerais, São Paulo (Fig. 15).

##### Host ant.

*Nomamyrmex esenbeckii* and *Nomamyrmex hartiigi*.

##### Remarks.

Borgmeier did not designate a holotype in his original description of the species, leaving four of the six specimens of the type series in his own collection and the others in the collections of Wasmann and Reichensperger. Among the four specimens retained by Borgmeier, two were identified as “Typus”, of which the best preserved one is designated here as the lectotype. For two other specimens, the information that they belong to the type series was found only on handwritten identification labels, as “paratypes”, and these are consequently regarded here as paralectotypes. Additionally, the type locality described as “Campinas, Goiás” by Schwarzmaier, refers to the city of Goiânia, “Campinas” being an old name for the city (Zara et al. 2007). The MZSP houses two female specimens identified as *E. spectabilis* with only paler coloration than the type specimens. In those specimens the enlarged flattened tubercles on tergites III–V are more evident (Fig. 4C) than in the type specimen (Fig. 4A), although the tubercles seem to be very similar to what can be observed in the photographs provided by Jenkins Shaw et al. (2023). This character was not used for comparison; instead, the aedeagus would likely be more useful in separating both species, although all studied specimens are females.

#### Ecitonides splendidus sp. nov.

Taxon classificationAnimaliaColeopteraStaphylinidae

57052376-A203-50C9-A7EB-46C618505BD7

https://zoobank.org/0C82E114-4428-4FA0-90CD-4F2D26040245

**Figure 13.**
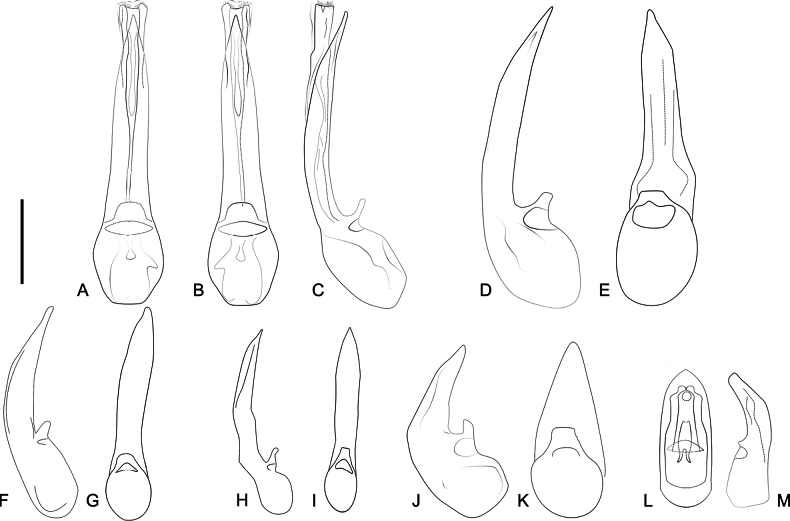
Aedeagi of *Ecitonides* species. **A–C**. *Ecitonides colossus* sp. nov.: **A**. Parameral view; **B**. Antiparameral view; **C**. Lateral view. **D, E**. *Ecitonides splendidus* sp. nov.: **D**. Lateral view; **E**. Antiparameral view. **F, G**. *Ecitonides tuberculosus*: **F**. Lateral view; **G**. Antiparameral view. **H, I**. *Ecitonides verrucosus*: **H**. Lateral view; **I**. Antiparameral view. **J, K**. *Ecitonides brevicornis*: **J**. Lateral view; **K**. Antiparameral view. **L, M**. *Ecitonides fraterculus*: **L**. Parameral view; **M**. Lateral view. Scale bars: 0.5 mm.

Figs 5, 9 B, I, 10D, 13D, E

##### Type locality.

Brazil • Maranhão: Mirador (Parque Estadual Mirador, Base da Geraldina), 06°37'25"S, 45°52'08"W, 12–20 Oct. 2012, F. Limeira-de-Oliveira, L. S. Santos and C.F. Barros leg.

##### Type specimen.

***Holotype*** (CZMA) male, dry pinned, with genitalia in a separate slide. Original labels: “Holótipo, *Ecitonides splendidus* Montanhini, Żyła and Biffi, 2025” // Brasil (MA), Mirador, Parque Est. Mirador, Base da Geraldina, 06°37'25"S/45°52'08"W // “Armadilha Luminosa, 12–20.x.2012, F. Limeira-de-Oliveira, L. S. Santos and C.F. Barros, cols.” // “CZMA Insect Collection” // “*Ecitonides* sp. nov. det. A. Asenjo, 2018”; “CZMA: FLO 15708”.

##### Diagnosis.

Body robust, head rectangular, pronotum hexagonal and flattened tubercles over the body as in *E. colossus* sp. nov., *E. constanceae* and *E. spectabilis*. It differs from other species by the labrum with five median denticles.

##### Description.

Body length: 9.5 mm. Body dark reddish-brown, antennae and tarsi slightly darker than body. Head, pronotum, elytra, tergites III and IV, and anterior margin of tergites V and VI covered with flattened tubercles, ventrally with rugose texture, with few distinct tubercles.

***Head***: elongate, ~ 2× as long as wide (HL: 1.8; HW: 0.8), rectangular, dorsal surface flat and covered with tubercles arranged in irregular longitudinal rows; ventral surface with distinct texture, with few distinct tubercles and covered with short, sparse, yellowish setae; frons as wide as posterior region; posterior margin only slightly converging. Eyes large, grayish, visible from above, occupying ~ 1/4 of lateral side of head. Antennae (Fig. 9B) with 11 antennomeres, elongate, twice longer than head, reaching posterior margin of pronotum; antennomeres moderately pubescent; scape as long as antennomeres 2–5 combined, with external margin sinuate, with apical emargination; antennomeres 2–10 longer than wide, 2 shorter than 3; 3 longer than 4–10; 4–10 slightly increasing in length; antennomere 11 subquadrate, apex acute. Labrum (Fig. 10D) well sclerotized, transverse, slightly emarginate and with five denticles; median tooth larger than others and pointed, with one pair of rounded denticles on each side, and adjacent pair of smaller denticles.

***Thorax***: pronotum (PL: 1.5; PW: 1.6) convex, hexagonal, widest at middle, wider than head; lateral margins diverging posteriorly at first half and converging posteriad and sinuate due to tuberculate sculpture; posterior margin sinuate, slightly wider than anterior margin; anterior margin feebly sinuate medially to accommodate small distinctive neck, as wide as 1/4 of maximum width of head. Pronotum covered with unequal rows of large tubercles with smaller tubercles in between, forming slight longitudinal crest. Prosternum without distinct tubercles, hypomera bearing small tubercles. Elytra (EL: 9; EW: 2.1) wider than pronotum, sinuate laterally and posteriorly due to tuberculate sculpture; anterior margin declivous; humeri weakly projected; each elytron with five regular longitudinal rows of small tubercles; each row marginated by small, thick setae. Meso- and metaventrite with rugose sculpture, with few conspicuous lateral tubercles and bearing short, thick setae; mesoventrite with small and shallow impression; mesosternal process acute and not reaching metasternal process; metasternal process small and truncate. Legs stout and relatively long, covered with small, sparse tubercles and short, thick setae; tarsomeres 1–4 transverse and subequal in size, tarsomere 5 longest, cylindrical, apically dilated, as long as previous four combined (Fig. 9I).

***Abdomen***: tergites III–V with posterior margin sinuate, more conspicuous in III and IV; VI with posterior margin slightly crenulated. Tergites III–IV with whole surface covered with tubercles and thick setae between; tergite V with distinct row of tubercles only at anterior 1/3 and flattened longer tubercles on posterior 2/3; VI without tuberculate sculpture, only impressions at base; VII with posterior margin straight, covered with small thick setae; VIII medially U–shaped and densely covered with short golden setae. Sternites III–VI with distinctive texture and covered with short, yellowish setae, laterally bearing thick, golden setae, outer margins weakly crenulated; VII covered with setae and slightly emarginate medially; VIII with median deep rounded emargination. **Aedeagus** (Fig. 13D, E) (LA: 1.6) long and slender, slightly flattened, bulbous at base, with truncate dorsal strut near foramen; apex of median lobe narrow and pointed, laterally curved.

**Figure 14.**
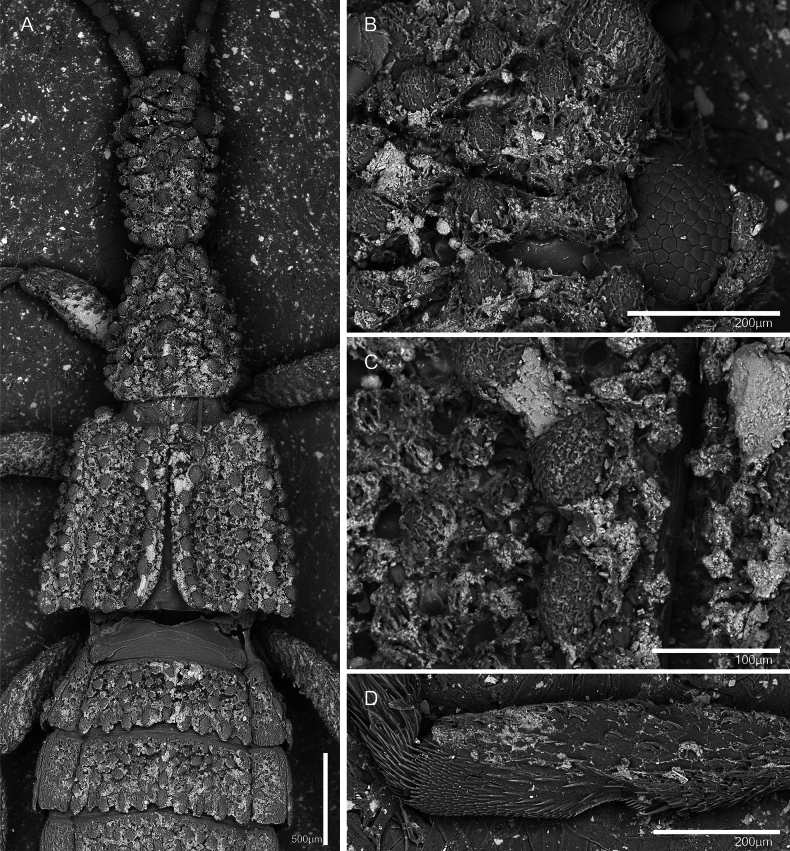
Scanning electron microscopy of the integument of *Ecitonides brevicornis* (NMW). **A**. Dorsal habitus; **B, C**. Details of the head and elytron showing the tubercles and adhered particles; the tubercles are covered with minute pubescence; **D**. Left fore tibia, showing detail of protibiae and the comb of setae.

**Figure 15.**
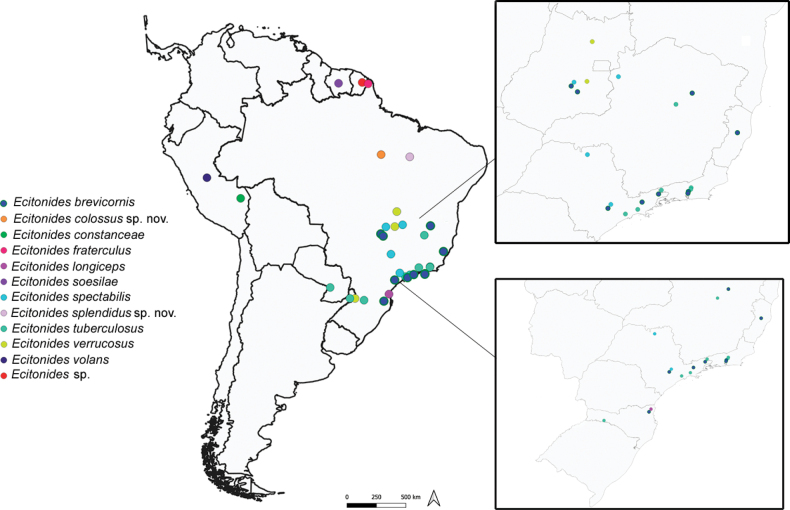
Distribution map of *Ecitonides* species.

##### Etymology.

The specific epithet splendidus is a Latin adjective, which means “splendid/bright”, in allusion to the bright coloration of the new species.

##### Distribution.

BRAZIL, Maranhão (Fig. 15).

##### Host ant.

Unknown.

#### Ecitonides tuberculosus

Taxon classificationAnimaliaColeopteraStaphylinidae

Wasmann, 1894

B5820C25-4E16-5DE7-B40E-70F3C0113BD3

Figs 6, 9 D, K, 10A, 11, 13F, G

Ecitonides tuberculosus Wasmann 1894: 212. Bruch 1933: 15 (redescription); Borgmeier 1949: 122.
*= Ecitonides fiebrigi* Wasmann, 1909: 182. Borgmeier 1949: 122 (syn).

##### Material examined.

Brazil (MZSP) • 1 undetermined sex; Minas Gerais; 16 Jun. 1923; E. luja leg.; MZSP21509 • 2 undetermined sex; Rio de Janeiro, Itatiaia; 5–9 Jul. 1932; J.F. Zikán leg • same collection data as for preceding; 25 Apr. 1935; MZSP 21504 • 1 ♂; 2 undetermined sex; same collection data as for preceding; MZSP 21503, 21505 • 1 ♀, 2 undetermined sex; same collection data as for preceding; Dirings leg; MZSP 21506, 21507 • 1 undetermined sex; São Paulo, Estrada Velha de Santos, Meio da Serra; 12 Feb. 1957; W. Kempf leg.; MZSP 21516 • 1 undetermined sex; São Paulo, Salesópolis, Estação Biológica de Boracéia; 25 Fev. 1963; Reichardt leg.; MZSP 21510 • 1 undetermined sex; same locality data as for preceding; 12–14 Nov. 1967; MZSP 21511 • 1 undetermined sex; same collection data as for preceding; 21–22 Mar. 1963; S. Vanin and M. Jorge leg.; MZSP 21512 • 1 undetermined sex; same collection data as for preceding; 3 Apr. 1973; A.S. Rano leg.; MZSP21513 • 1 ♂; same collection data as for preceding; 14–15 Apr. 2023; H.P. Moleiro leg.; MZSP 21517 • 1 undetermined sex; Santa Catarina, Nova Teutônia; Oct. 1936; F. Plaumann leg.; MZSP 21514; BRAZIL (CEMT) • 1 ♂, 1 ♀; Rio de Janeiro, Nova Friburgo; Nov. 2009; E. Grossi leg • 1 undetermined sex; same locality and collector data as for preceding; Mar. 2012.

##### Diagnosis.

Body less robust, head oval, pronotum longer than wide and subconical, and prominent tubercles over body as in *E. brevicornis*, *E. fraterculus*, *E. longiceps*, *E. volans*, and *E. soesilae*. From *E. brevicornis* and *E. fraterculus*, it differs by antennae being almost twice as long as head (same length as head in other two species), from *E. longiceps* by larger eyes, from *E. volans* by pattern of the tuberculate sculpture on tergites III–V, which is denser in *E. volans* and sparser in *E. tuberculosus*, and from *E. soesilae* by each elytron with four regular rows of tubercles (5 rows in *E. soesilae*).

##### Additional characters.

Antennae (Fig. 9D) with scape almost as long as antennomeres 2–6 combined; antennomere 2 slightly shorter than 3; antennomeres 3–11 longer than wide and of subequal length; labrum (Fig. 10A) with three small denticles: one median, acute, and one rounded denticle on each side; abdomen with tergites III–V and paratergites of segments III and IV bearing small tubercles; sternites III and IV bearing small and flattened tubercles and III–V with ridges extending beyond posterior margin. Aedeagus (Fig. 13F, G) with rounded dorsal strut near foramen; apex of median lobe narrow and pointed, weakly sinuate.

##### Measurements.

TL: 6.0–7.5; HL: 1.51; HW: 0.78; PL: 1.2; PW 0.8: EL: 1.41; EW: 1.32; LA: 1.1.

##### Distribution.

BRAZIL, Minas Gerais, Rio de Janeiro, São Paulo, Santa Catarina. PARAGUAY, Cordillera. ARGENTINA, Misiones (Fig. 15).

##### Host ant.

*Labidus praedator*.

##### Remarks.

Most of the studied specimens are old, entirely glued to cardboards, and therefore fragile. In order to preserve them, it was decided not to unglue them, making sex determination impossible.

Borgmeier (1949) synonymized *E. fiebrigi* with *E. tuberculosus* because the size difference (7.5 mm and 6.0 mm, respectively) was considered insufficient to separate them. Although the description and illustrations given for *E. tuberculosus* enable reliable identification of additional material from Brazilian collections, no illustrations were given for *E. fiebrigi*, and the description is insufficient to clearly distinguish the two species. The confirmation of the synonymy will require comparison of the type specimens of both species, which were not available to us.

#### Ecitonides verrucosus

Taxon classificationAnimaliaColeopteraStaphylinidae

Bruch, 1933

E2631584-8CCC-534B-B533-E126F0487D21

Figs 7, 9 G, 10G, 12A, D, 13H, I

Ecitonides verrucosus Bruch 1933: 12; Borgmeier 1949: 122 (redescription and notes).

##### Material examined.

Brazil (MZSP) • 1 ♂; Goiás, Niquelândia; 24. Sep. – 06 Oct. 1995; Silvestre, Dietz, and Brandão leg.; MZSP 21500 • 1 ♀; Goiás, Leopoldo Bulhões; 30 Oct. 1933; Schwarzmaier leg; MZSP 21501.

##### Diagnosis.

*E. verrucosus* differs from all other species by distinctive sparser pattern of tubercles on head and pronotum, forming distinct rows on head and pronotum, head oblong and wider posteriorly, pronotum trapezoidal and shorter than head.

##### Additional characters.

Tubercles prominent, antennae (Fig. 9G) ~ 1.5× longer than head, scape as long as antennomeres 2–6 combined, antennomere 2 longer than 3, antennomeres 3–10 subquadrate, antennomere 11 longer than 10; labrum (Fig. 10G) with two rounded lateral denticles, without median one; elytra with four rows of tubercles; abdomen with tergites and sternites III–VI with ridges extending beyond posterior margin, without distinct tubercles; aedeagus (Fig. 13H, I) with median lobe slightly sinuate, with rounded and sinuate dorsal strut near foramen; apex of median lobe narrow and pointed.

##### Measurements:

TL: 5.0; HL: 1.21; HW: 0.68; PL: 0.84; PW: 0.78; EL: 1.19; EW: 1.44; LA: 1.0.

##### Distribution.

ARGENTINA, Misiones. BRAZIL, Goiás (Fig. 15).

##### Host ant.

*Labidus coecus*.

##### Remarks.

This species is known from Argentina. One of the specimens deposited in the MZSP collection was identified by Borgmeier; however, the specimen illustrated here (Fig. 7) was identified primarily based on the original description, as the specimen identified by Borgmeier is in poor condition. The identity of the illustrated specimen remains to be confirmed through comparison with the type specimens.

#### Ecitonides volans

Taxon classificationAnimaliaColeopteraStaphylinidae

Assing, 2012

6D48783F-CE4E-58E3-9434-030A71CF5299

Fig. 8

Ecitonides volans Assing 2012: 402.

##### Type locality.

Peru • Huanuco: Panguana station at Rio Llullapichis, 9°37'S, 74°56'W, 260 m, G. Riedel leg.

##### Type specimen examined.

***Holotype*** (NMW) male, dry pinned. Original labels: “Peru, Huanuco Prov., Panguana station at Rio Llullapichis, 9°37'S, 74°56'W, 260 m, at light, 2.-20.X.2009, leg. G. Riedel” // “Holotypus *Ecitonides volans* sp.n. det. V. Assing 2012”.

##### Diagnosis.

Body less robust, head oval, pronotum longer than wide and subconical, and prominent tubercles over body as in *E. brevicornis*, *E. fraterculus*, *E. longiceps*, *E. soesilae*, and *E. tuberculosus*. From *E. brevicornis* and *E. fraterculus*, it differs by each elytron with five rows of tubercles (4 in *E. brevicornis* and 3 in *E. fraterculus*). From *E. longiceps* by larger eyes, from *E. soesilae* by denser tuberculate sculpture (with tubercles prominent and sparser and with tubercles slightly flattened in *E. soesilae*), and from *E. tuberculosus* by antennae of same length as head (almost twice longer than head in *E. tuberculosus*).

##### Additional characters.

Antennae with scape as long as antennomeres 2–4 combined; antennomeres 2 and 3 subequal in size; antennomeres 4–10 longer than wide, of subequal size; antennomere 11 shorter than 10. Abdomen with tergites III–V and paratergites of III and IV with large tubercles.

##### Distribution.

PERU, Huanuco (Fig. 15).

##### Host ant.

Unknown.

### Additional material examined of *Ecitonides*

A specimen from French Guiana, labelled as “*Ecitonides sp. aff. volans*” and identified by V. Assing, is present in the NHMUK collection. Due to its poor condition, the identification could not be confirmed with certainty. Nevertheless, it represents the first record of the genus from French Guiana, expanding its known distribution to six countries (Fig. 15).

FRENCH GUYANA (NHMUK) • 1 ♀; “Station des Nouragues, Saut Pararé, 4°02'N, 52°41'W, 65m; Sep. 2009”.

## Discussion

The genus *Ecitonides* represents a group of Neotropical myrmecophilous rove beetles that are both morphologically distinctive and rarely collected. Despite being known for more than a century, most species remain poorly characterized, with incomplete diagnoses, insufficient illustrations, and little information on their biology and distributions. In this study, we provide new morphological data, describe two species new to science, and update the geographic distribution for the genus. These results not only expand our knowledge of *Ecitonides* diversity but also provide a foundation for future studies on their systematics, phylogeny, and biogeography.

The eleven currently recognized species of *Ecitonides* can be divided into three different species groups, based on external morphological features:

(1) *E. spectabilis*, *E. constanceae*, *E. colossus* sp. nov., and *E. splendidus* sp. nov. share a more robust body, a parallel-sided head, a pronotum slightly longer than wide to hexagonal and flattened tubercles over the body. *Ecitonides colossus* sp. nov. can be distinguished from the other species of the group by the reduced eye size. *Ecitonides spectabilis* can be distinguished from *E. constanceae* by the size and shape of eyes, which are larger and distinctly interrupting the outline of the head in dorsal view in *E. constanceae* (Jenkins Shaw et al. 2023). It can be further separated from *E. splendidus* sp. nov. by the wider body, a pronotum that is slightly longer than wide (hexagonal in *E. splendidus* sp. nov.), and by the labrum, which has three denticles in *E. spectabilis*, while in the new species there are five.

(2) *E. tuberculosus*, *E. brevicornis*, *E. longiceps*, *E. fraterculus*, *E. volans*, and *E. soesilae* share a less robust body, an oval head, a pronotum longer than wide and subconical, prominent tubercles over the body. *Ecitonides longiceps* can be distinguished from the other species of this group by the reduced eyes. *Ecitonides brevicornis* is more similar to *E. fraterculus*, but it is distinguishable by the shape of antennomeres 6–10, which are as long as wide in *E. brevicornis* and clearly transverse in *E. fraterculus*, and by the number of regular rows of tubercles on each elytron (four in *E. brevicornis* and three in *E. fraterculus*). *Ecitonides tuberculosus* can be easily distinguished from *E. volans* by the pattern of the tuberculate sculpture on tergites III–V, which is denser in *E. volans* (Fig. 8).

(3) *E. verrucosus* has unique features and can be distinguished from all other species by the very sparse and distinctively patterned tuberculate sculpture, the oval head posteriorly wider than anteriorly, and the pronotum of trapezoidal shape.

*Ecitonides* differs from other genera with a distinctive tuberculate sculpture (*Bolbophites*, *Synecitonides*) by the shape of the head and the structure of the setigerous tubercles. In *Synecitonides* and *Bolbophites*, the posterior margin of the head is ~ 1/2 as wide as the frons, gradually narrowing towards the neck, and the head is widest between the eyes. In *Ecitonides*, the posterior margin is at least 3/4 as wide as the frons, feebly convergent, with rounded posterior angles, and the head is widest between the eyes or posteriorly. The genera also differ in the size, shape, and distribution pattern of the tubercles: in *Ecitonides*, they are larger, either prominent or flattened, and densely distributed on the head and pronotum; in *Synecitonides* and *Bolbophites*, the tubercles are smaller, uniformly prominent, arranged in only a few regular rows, and in *Bolbophites* they are absent from the head.

Since the original description of the genus, different authors have proposed affinities with genera such as *Echiaster* and *Myrmecosaurus* based on characters including head shape, tuberculate sculpture, and elongate tarsomeres. Seevers (1965) grouped *Ecitonides* with seven related genera in the “*Ecitonides*-group” defined by shared ornamentation of tubercles, carinae, and grooves, while later authors suggested placement in Echiasterina or even Cryptobiina, relying on mouthparts, antennae, and male sexual characters. More recently, Schomann and Solodovnikov (2017) placed the genus in Lathrobiini, and Jenkins Shaw et al. (2023), through molecular data, recovered it within the “Medonina and allied taxa clade”. However, these hypotheses are based either on limited morphological comparisons or on molecular analyses with restricted gene and taxon sampling. Even though all species of the ‘*Ecitonides*-group’ share similar characters, most likely convergences resulting from myrmecophily, they do not necessarily constitute a natural group. This underscores the need for a comprehensive morphological and molecular study of *Ecitonides* species and related genera to clarify their phylogenetic placement.

Due to limited information on species distribution, primarily resulting from the low number of collected specimens, it remains difficult to assess how geographically restricted some species may be. The genus is now recorded in six countries (Argentina, Brazil, French Guiana, Paraguay, Peru and Suriname), occurring in habitats such as the Brazilian Savanna, the Atlantic Forest, and the Amazon – all species with more than two records and with identified associated ants are reported from Cerrado, Atlantic Forest, and/or transitional areas between them, but never restricted to a single habitat type. The new record from French Guiana thus extends the northern distributional limit of the genus within the Amazonian region.

Furthermore, there are no illustrations or photographs of the aedeagi, and, except for *E. tuberculosus* and *E. verrucosus*, all *Ecitonides* species seem to have very restricted distribution. A similar issue applies to the specimen of *E. tuberculosus* described and illustrated by Bruch (1933) from Argentina, although the description is detailed, it does not provide sufficient evidence to confirm whether these specimens actually belong to the same species or not. According to Seevers (1965), *Labidus praedator* (Smith, 1858) is the army ant species that harbors the greatest number of guests, and he suggested that the isolation of its populations may have provided multiple opportunities for speciation, with hosts and their symbionts evolving together in isolation. Seevers (1965) reasoning, originally proposed to explain the remarkable diversity of myrmecophiles associated with *L. praedator*, can also be extended to other *Ecitonides* hosts, since all of them have a very wide range distribution across the Americas, ranging from the southern United States to Argentina. Therefore, it is plausible that the distribution of the genus is broader than currently documented.

### Host relationships and morphological adaptations

Most species of Staphylinidae living within colonies of army ants are rare or at least uncommon (Akre and Rettenmeyer 1966). Although the biology of the genus is poorly known, the host ant species is known for most of the species. *Labidus praedator* is the host for *E. brevicornis*, *E. fraterculus* and *E. tuberculosus*, *Labidus coecus* (Latreille, 1802) is the known host for *E. verrucosus* and *E. longiceps*, and *Nomamyrmex esenbeckii* (Westwood, 1842) and *Nomamyrmex hartiigi* (Westwood, 1842) are recorded as the hosts of *E. spectabilis* (Seevers 1965) (Table 1). Wasmann (1894) originally described *Eciton quadriglume* (Haliday, 1836) as the host of *E. tuberculosus*, but in 1900 he corrected the identification of the ant to *L. praedator*. The host ants of the recently described species, *E. volans*, *E. soesilae* and *E. constanceae*, as well as the two new species described here, which were collected mostly in light traps, are unknown. However, for the localities of the two new species, there are records for three species of the host ants, except for *N. hartiigi* in Maranhão (Prado et al. 2019; de Albuquerque et al. 2021).

Wasmann (1900) suggested that the mouthparts of *Ecitonides* may indicate that species of the genus are predators of *Labidus* (by that time, *Eciton* Latreille, 1804), although he also considered possible the existence of a more intimate relationship between the genus and its hosts due to the prominences referred to as tubercles. In 1909, he briefly reported that the specimen described as *E. fiebrigi* was collected in a prey deposit of army ants, similar to Bruch’s (1933) observation for *E. verrucosus*. These reports also indicate that the species of the genus are probably well integrated into the ant colonies and feed on the prey of the host ants rather than predating on the ants. Species inhabiting these deposits appear to have developed a stronger association with the ants, potentially depending on their presence to complete their lifecycles (von Beeren et al. 2023).

Although Wasmann (1900) did not perform histological analyses, he suggested that the tubercles replace trichomes because the surface is apparently glabrous. However, at 20× magnification, short setae on tubercles are clearly visible (Fig. 14B, C) and, in the largest species (e.g., *E. spectabilis*, *E. colossus* and *E. splendidus*) short setae are visible even without high magnifications (Fig. 2A, C). Assing (2012) suggested that these tubercles are probably modified setae rather than modifications of the exoskeleton. Presumably, as the genus is well integrated and assimilated into colony life, those structures might be a modification of the integument for worker ants to pick up the inquilines and carry them around, as observed for other myrmecophiles (Parker 2016).

Guests of army ants usually closely resemble their hosts in coloration and other integumental specializations (Seevers 1965). The resemblance in color possibly represents an adaptive response to visual predators. However, for species associated with subterranean army ants, differences between the body coloration of the hosts and inquilines might be a sign that other selection pressures are acting (von Beeren et al. 2018). When describing *E. fiebrigi*, Wasmann (1909) suggested that traits such as coloration, elongation of head and eyes size are likely the result of the myrmecophilous lifestyle. At that time, only three species were known, and he noted the high contrast between the coloration of the species of the genus and its host ants. He mentioned that the reduced eyes size and longer head of *E. longiceps* might be a result of the association with *L. coecus* as this species has rudimentary and less developed eyes than of *L. praedator*. Borgmeier (1932, 1949) also discussed the discrepancy of coloration of host ants and their guests for these species, and described *E. spectabilis*, whose body color matches the host. Later, he argued that the proportions of the head, which in *E. longiceps* is actually shorter than it was originally described, cannot alone justify myrmecophily or classify the genus as a “mimic-type”, considering that the genus has no similarity to ants. Also, the association between the reduced eyes of *E. longiceps* and its hosts (*L. coecus*), might not be directly related to the relationship between these species, since *E. verrucosus*, which has larger eyes and other distinctive features, is also a guest of *L. coecus*.

In the same revision, Borgmeier discussed the conspicuous constriction present on pro- and mesotibiae that might be an adaptation to the myrmecophily, facilitating better attachment to the host, similar to what is observed in *Mimophites*. He also noted that the grayish-yellow color is mostly caused by adhered particles (Fig. 14B, C), as the true body coloration is dark (brown to rust-red), unless the individuals are teneral. Species deposited in MZSP are mostly the same ones Borgmeier used in his revision in 1949, and in most species, there are some visible color variations apart from adhered particles. For example, some specimens of *E. tuberculosus* appear grayish-yellow, apparently due to the adhered soil particles, although there are specimens of the same species, mostly collected in light traps, that are naturally paler. In *E. spectabilis*, darker individuals include almost black individuals (Fig. 4A) clearly covered with adhered particles, as well as a few pale reddish-brown ones (Fig. 3C); in both cases, the true coloration did not change when the particles were removed.

Although myrmecophilous Paederinae remains a fascinating yet poorly known group, *Ecitonides* stands out as a particularly intriguing genus. Its diversity, geographic distribution, biology, and phylogenetic position are still incompletely understood, and many aspects of its morphology, host associations, and ecological adaptations remain to be explored. The new species described here, with updated distribution records and morphological data, highlight the potential for undiscovered diversity and complex evolutionary relationships within the genus. Future studies combining detailed morphological, molecular, and ecological approaches are essential to understand the evolutionary history of *Ecitonides* and the mechanisms shaping its associations with army ants.

## Supplementary Material

XML Treatment for Ecitonides

XML Treatment for Ecitonides brevicornis

XML Treatment for Ecitonides colossus

XML Treatment for Ecitonides fraterculus

XML Treatment for Ecitonides spectabilis

XML Treatment for Ecitonides splendidus

XML Treatment for Ecitonides tuberculosus

XML Treatment for Ecitonides verrucosus

XML Treatment for Ecitonides volans

## Additional information

### Use of AI

No use of AI was reported.

### Author ORCIDs

Amanda Montanhini https://orcid.org/0009-0006-3597-8860

Dagmara Żyła https://orcid.org/0000-0003-4959-763X

Gabriel Biffi https://orcid.org/0000-0001-7532-3822

### Data availability

All of the data that support the findings of this study are available in the main text.
